# Evaluating the Effectiveness of Nivolumab in Metastatic Lung Cancer Among Patients Aged 65 and Older

**DOI:** 10.3390/jcm13206263

**Published:** 2024-10-20

**Authors:** Deniz Isik, Özkan Alan, Goncagül Akdağ, Sedat Yildirim, Oğuzcan Kınıkoğlu, Yunus Emre Altintas, Ezgi Turkoglu, Heves Surmeli, Tugba Basoglu, Ozlem Nuray Sever, Hatice Odabas, Mahmut Emre Yildirim, Nedim Turan

**Affiliations:** 1 Department of Medical Oncology, Health Science University, Kartal Dr. Lütfi Kirdar City Hospital, Istanbul 34865, Türkiye; akdaggoncagul@gmail.com (G.A.); rezansedat@hotmail.com (S.Y.); ogokinikoglu@yahoo.com (O.K.); yunusaltintas1688@gmail.com (Y.E.A.); ezgiturk_90@hotmail.com (E.T.); hevessurmeli@hotmail.com (H.S.); basoglutugba@gmail.com (T.B.); ozlem.sever@hotmail.com (O.N.S.); odabashatice@yahoo.com (H.O.); emremahmutyildirim@gmail.com (M.E.Y.); turan.nedim@hotmail.com (N.T.); 2 Division of Medical Oncology, School of Medicine, Koç University, Istanbul 34450, Türkiye; ozkan.alan@hotmail.com

**Keywords:** non-small cell lung cancer, nivolumab, older patients

## Abstract

**Background:** Lung cancer remains the leading cause of cancer-related mortality globally, predominantly affecting older individuals. Despite the increasing use of immune checkpoint inhibitors (ICIs) like nivolumab in non-small cell lung cancer (NSCLC), the efficacy and safety in elderly patients, particularly those aged 65 and above, remain underexplored due to their underrepresentation in clinical trials. **Methods:** This retrospective study analyzed data from 60 elderly patients (≥65 years) with metastatic NSCLC who received nivolumab as second-line or later therapy between January 2020 and May 2023. **Results:** The median age was 67 years, with a predominance of males (78%). Nivolumab was administered for a median of 8 cycles, with 33.3% of patients receiving 15 or more cycles. The median OS was 23 months, and the 1-, 3-, and 5-year survival rates were 93.3, 54.1, and 18.6%, respectively. Multivariate analysis identified adenocarcinoma histology, fewer than 15 cycles of nivolumab, and non-response to prior therapies as independent predictors of poor OS. Nivolumab treatment was generally well-tolerated, with 45% of patients experiencing at least grade 1 toxicity. **Conclusions:** Nivolumab is effective and well-tolerated in elderly patients with metastatic NSCLC, providing survival benefits comparable to those observed in younger populations. The number of treatment cycles and initial response to therapy are key determinants of survival, underscoring the importance of continued treatment in this age group.

## 1. Introduction

Lung cancer is the third most common type of cancer globally and the leading cause of cancer-related mortality in both sexes [1]. The average age at diagnosis is 71 years, with most cases being diagnosed between 65 and 74 [2]. As a disease predominantly affecting older individuals, lung cancer treatment presents significant challenges due to comorbid conditions, declining organ function, disorientation, and limited social support. Despite older patients with lung cancer comprising a substantial portion of our daily clinical practice, their underrepresentation in clinical trials has led to limited knowledge regarding treatment efficacy and adverse effects in this population. For instance, a review by the FDA reported that only 24% of patients in cancer clinical trials conducted between 2005 and 2015 were aged 70 or older [3].

Over the past decade, immunotherapy has revolutionized the treatment of various cancers, particularly lung cancer, malignant melanoma, and renal cell carcinoma. The low response rates and high toxicity of chemotherapy in lung cancer, along with its significant impact on quality of life and the limited presence of targetable mutations in a small subset of patients, have shifted the focus toward immunotherapy as a primary treatment option for lung cancer [4]. Specifically, immune checkpoint inhibitors (ICIs), which target PD-1 and PD-L1 inhibition, have become a standard second-line treatment for patients with non-small cell lung cancer (NSCLC) after demonstrating a survival advantage over monotherapy with single-agent chemotherapy [5,6,7,8]. Currently, ICIs are used in both monotherapy and combination with chemotherapy in neoadjuvant and adjuvant settings, as maintenance therapy following definitive chemoradiotherapy, or as first-line treatment in metastatic disease. These therapies enhance the immune system’s ability to mount an anti-tumor response. However, the efficacy and safety of ICIs in older populations, particularly in the context of immunosenescence—a decline in innate and adaptive immunity with age—remain unclear.

In this study, we aimed to evaluate the efficacy and safety of immunotherapy in elderly patients, defined as those aged 65 and above, diagnosed with metastatic NSCLC.

## 2. Materials and Methods

### 2.1. Study Design and Data Collection

In this study, we retrospectively analyzed data from patients 65 years and older diagnosed with NSCLC who received at least one cycle of nivolumab between January 2020 and May 2023. Inclusion criteria were (1) a histologically confirmed diagnosis of NSCLC; (2) age 65 or older at the initiation of nivolumab; and (3) receipt of at least one cycle of nivolumab. 

Before entering the study, all patients received physical examination, complete blood count, and serum chemistry analyses. Positron emission tomography–computerized tomography (PET-CT) ( General Electric Healtcare, Amersham, United Kingdom) was used in these cases. During treatment, complete blood count and serum chemistry analyses were conducted before each cycle, and PET-CT scans were conducted every six cycles. Responses were evaluated according to iRRECIST criteria. Toxicities were graded according to the National Cancer Institute of Common Toxicity Criteria (version 5.0). The primary endpoint of this study was OS and PFS. The secondary endpoint was the toxicity profile. Patients were considered assessable for response if they received at least one cycle of immunotherapy. Safety analyses included all treated patients and involved analyzing treatment-emergent adverse events.

### 2.2. Statistical Analysis

This study tested the normality assumption of continuous variables using the Shapiro–Wilk-W test. Categorical variables were presented as frequencies (*n*, %), while continuous variables were reported as medians and the Interquartile Range (IQR). Comparisons between categorical variables were performed using Pearson and Fisher’s exact chi-square tests. For analysis, certain continuous variables (age, BMI, and NLR) were transformed into categorical variables based on their median and quartile distribution. The optimal IT cut-off value for progression-free survival was determined using the Cox proportional hazards regression model. Overall and progression-free survival times were calculated using the Kaplan–Meier method. Differences between the calculated survival curves were compared using the log-rank test. Univariate and multivariate Cox regression models were utilized to identify variables affecting overall and progression-free survival. Results were considered significant at a 95% confidence interval with a *p*-value of less than 0.05 (two-sided). All statistical analyses were conducted using SPSS version 27 (IBM Corp., Armonk, NY, USA) and R statistical software (version 4.4.1; www.r-project.org. Accessed on 17 July 2024).

## 3. Results

### 3.1. Patient Characteristics

A total of 60 patients aged 65 years and older diagnosed with lung cancer were included in the study. The median age was 67 years (IQR, 66–71; range, 65–88), and 47 patients (78%) were male. Seventy percent of the patients had an additional chronic condition alongside their current illness. Histologically, the tumor type was classified as adenocarcinoma in 27 patients (45%), squamous cell carcinoma in 22 patients (36.7%), and mixed type in 11 patients (18.3%). At the time of diagnosis, 65% of the patients had metastatic disease. Nivolumab was administered as second-line therapy in 44 patients (73.3%) and third or fourth-line therapy in 16 patients (26.7%). During treatment, 27 patients (45%) experienced at least grade 1 toxicity; however, no mortality related to toxicity was observed. Nivolumab was administered for fewer than 15 cycles in 40 patients (66.7%), while 20 patients (33.3%) received 15 or more cycles. Post-treatment evaluations showed a partial or complete response to treatment in 22 patients (36.7%). Detailed demographic characteristics of the patients are presented in Table 1.

### 3.2. Overall Survival and Analysis of Influencing Factors

During the median follow-up period of 23 months (IQR, 19–36), mortality was observed in 32 patients (53.3%) (Table 2). The overall survival probabilities at 1, 3, and 5 years were calculated as 93.3%, 54.1%, and 18.6%, respectively. In univariate analyses, patients with multiple organ metastases [HR, 2.10 (95% CI, 1.03–4.31); *p* = 0.042], those who did not respond to prior systemic therapies [HR, 2.43 (95% CI, 1.05–5.62); *p* = 0.039], and those who did not respond to nivolumab [HR, 3.65 (95% CI, 1.05–5.62); *p* = 0.004] had significantly worse overall survival. In contrast, patients who completed 15 or more cycles of nivolumab [HR, 0.07 (95% CI, 0.02–0.29); *p* < 0.001] had better survival outcomes (Table 2).

**Figure 1:**
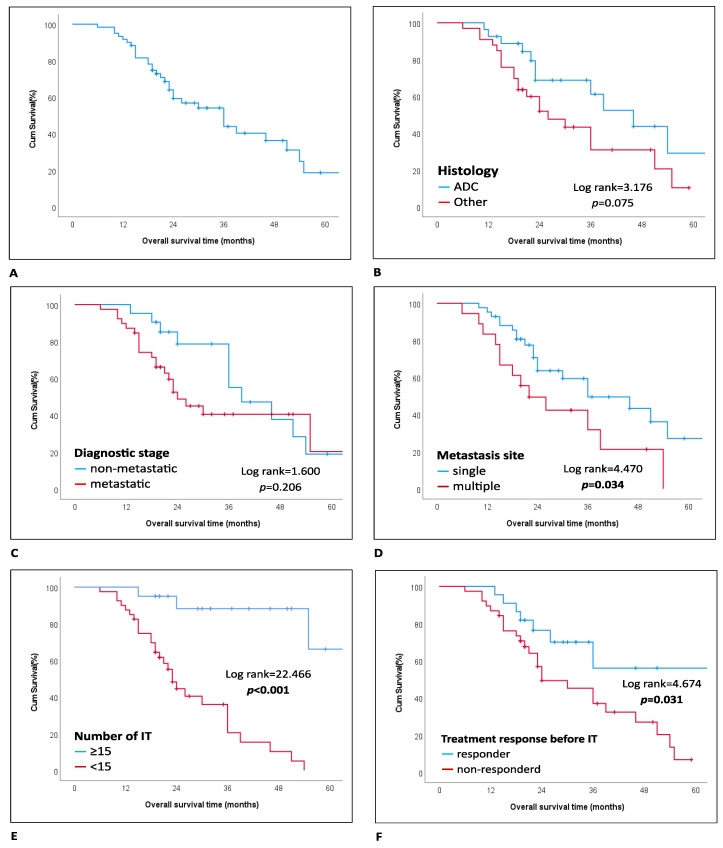
Kaplan–Meier curve showing factors significantly affecting overall survival. (**A**): Kaplan–Meier curve showing overall survival. (**B**): Comparison of overall survival between patients with adenocarcinoma (Adenoca) and other histologies. (**C**): Kaplan–Meier curves comparing overall survival in patients diagnosed at non-metastatic versus metastatic stages. (**D**): Single vs. multiple organ metastases and their impact on overall survival. (**E**): Comparison of overall survival based on the number of IT cycles (<15 vs. ≥15). (**F**): Analysis of overall survival in patients classified as responders or non-responders to prior chemotherapy or radiotherapy.

Multivariate analysis identified several independent variables associated with poorer overall survival: tumor histology other than adenocarcinoma [HR, 5.31 (95% CI, 1.74–16.18); *p* = 0.003], metastatic status at diagnosis [HR, 13.43 (95% CI, 3.69–48.96); *p* < 0.001], lack of response to prior systemic therapy [HR, 4.50 (95% CI, 1.16–17.55); *p* = 0.030], and no response to nivolumab [HR, 6.89 (95% CI, 1.30–36.56); *p* = 0.023]. Additionally, completion of 15 or more cycles of nivolumab was associated with a significant positive impact on overall survival [HR, 0.09 (95% CI, 0.01–0.74); *p* = 0.024] (Figure 1).

### 3.3. Progression-Free Survival and Analysis of Influencing Factors

**Figure 2:**
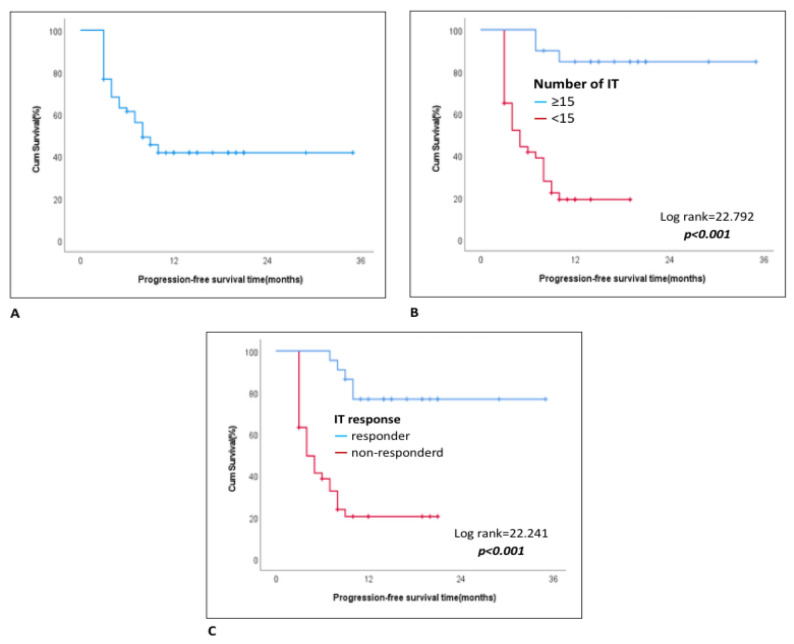
Kaplan–Meier curve showing factors significantly affecting progression-free survival. (**A**): Kaplan–Meier curve showing progression-free survival. (**B**): Kaplan–Meier curves comparing progression-free survival between patients receiving fewer than 15 immunotherapy (IT) cycles and those receiving 15 or more cycles. (**C**): Kaplan–Meier analysis of PFS based on the patient’s response to immunotherapy.

The median progression-free survival for patients receiving nivolumab was 8 months (IQR, 3–14) (Table 3). The progression-free survival probabilities at 1, 2, and 3 years were 69.2, 8.1, and 4.1%, respectively. Both univariate and multivariate analyses revealed that a lack of response to nivolumab was associated with decreased progression-free survival [HR, 6.85 (95% CI, 2.59–18.08); *p* < 0.001 and HR, 7.76 (95% CI, 2.11–28.61); *p* = 0.002], whereas completing 15 or more cycles of treatment was associated with increased progression-free survival [HR, 0.10 (95% CI, 0.03–0.34); *p* < 0.001 and HR, 0.13 (95% CI, 0.03–0.54); *p* = 0.005] (Figure 2).

### 3.4. Treatment Response and Associated Variables

Clinical and radiological evaluations following immunotherapy showed that 3 patients (5%) achieved a complete response, 19 patients (31.7%) had a partial response, 15 patients (25%) had stable disease, and 23 patients (38.3%) experienced disease progression. The only variable significantly associated with treatment response was the number of treatment cycles. Among patients who received fewer than 15 cycles, 15% achieved a complete or partial response, whereas 80% of those who received 15 or more cycles responded to treatment. Compared to patients who received fewer than 15 cycles, those who completed 15 or more cycles of nivolumab had a 22.7-fold increase in treatment response (95% CI, 5.6–91.7; *p* < 0.001) (Table 4).

## 4. Discussion

Lung cancer is generally considered a disease of older age, with approximately 70% of patients being 65 years or older at the time of initial diagnosis [2]. Despite being the most common age group in our daily practice, older patients, particularly those aged 70 and above, are significantly underrepresented in clinical trials due to various organ dysfunctions and comorbid conditions [3]. Additionally, the elderly population included in clinical trials tends to be fitter than the general older population, leading to a lack of experience and knowledge, particularly concerning the treatment of frail, elderly patients.

Since 2016, immunotherapies, particularly immune checkpoint inhibitors (ICIs), have revolutionized modern cancer treatment. In lung cancer, ICIs have become central to treatment, whether used as monotherapy or in combination with chemotherapy. These agents offer significant overall survival advantages compared to chemotherapy and have a better safety profile. However, the potential for rare but severe immune-related adverse events, which can be more challenging to manage in elderly and frail patients, raises concerns about their use in this population.

The World Health Organization (WHO) classifies individuals aged 65 and older as elderly, a group whose functional capabilities need to be supported and enhanced [9]. In clinical trials, patients aged 65 and above are typically considered part of the elderly subgroup. Despite these classifications, it is crucial to distinguish between biological age—defined by comorbidities, frailty, and life expectancy—and chronological age when planning treatment. Aging is associated with changes in the immune system, including thymic involution, reduced hematopoiesis, and increased B memory cells, collectively known as immunosenescence [10]. These changes make it unclear how older patients, particularly those receiving immunotherapy, will respond to cancer treatments.

In our study, we analyzed data from 60 patients who received nivolumab as a second- or later-line therapy, which is the standard treatment in our country due to reimbursement policies. The progression-free survival (PFS) of 8 months observed in our study is consistent with real-world data from the literature. We found a statistically significant PFS interval in patients who received 15 or more cycles of immunotherapy. In terms of overall survival (OS), the number of immunotherapy cycles was again significant, with patients with adenocarcinoma histology surviving significantly longer. Our 5-year OS rate of 18.6% is consistent with the literature. These findings suggest that nivolumab treatment in elderly patients is as effective as in younger patients. However, 45% of our patients experienced toxicity of any grade related to immunotherapy. Although 40% of our patients experienced treatment delays due to side effects, no treatment-related mortality was observed.

In phase 3 pivotal immunotherapy trials involving patients with metastatic lung cancer, the representation of those aged 65 and older was only 41–55%. The efficacy of immunotherapy was first demonstrated in metastatic non-small cell lung cancer (NSCLC) patients who had received at least one line of chemotherapy and subsequently experienced disease progression. Nivolumab was the first drug in this class to show efficacy. In the CheckMate 017 trial, 131 patients with metastatic squamous cell lung cancer who had previously received chemotherapy were randomized to receive nivolumab, and 129 patients were randomized to receive docetaxel. Nivolumab was associated with a 41% reduction in the risk of death (HR: 0.59; 95% CI, 0.44–0.79) [5]. Of these patients, 91 (33%) were aged 65–74, and this group also showed a 44% reduction in the risk of death, similar to the overall study population (HR: 0.56; 95% CI, 0.34–0.91). In the similarly designed CheckMate 057 trial, which included non-squamous lung cancer patients, 287 patients were randomized to receive nivolumab and 268 to docetaxel [6]. Nivolumab demonstrated a 27% advantage in reducing the risk of death compared to docetaxel (HR: 0.73; 95% CI, 0.59–0.89). This study included 200 patients aged 65–74 (34%), who also experienced a 37% reduction in the risk of death (HR: 0.63; 95% CI, 0.45–0.89). While the CM-017 and CM-057 trials demonstrated survival benefits for the 65–74 age group, these advantages were either diminished or lost entirely in patients aged 75 and older. In the CM-057 trial, the HR for patients over 75 was 0.90, while in the CM-017 trial, it was 1.85. Due to the small number of patients in this age group, accounting for only 11% of the total trial population, further studies are needed to determine whether this is detrimental. A combined analysis of these two trials also showed a survival benefit for nivolumab in both the under-65 (HR: 0.66) and over-65 (HR: 0.71) age groups after five years of follow-up [11].

Pembrolizumab, another ICI, was compared to docetaxel in the Keynote-010 trial in patients with metastatic NSCLC who had received at least one line of chemotherapy and had a PD-L1 expression level of 1% or higher [8]. This three-arm phase 2 trial included 1034 patients, with pembrolizumab administered at 2 mg/kg and 10 mg/kg. Pembrolizumab showed a survival advantage over docetaxel in the general patient population, with an HR of 0.71 (95% CI, 0.58–0.88) for the 2 mg/kg dose and an HR of 0.61 (95% CI, 0.49–0.75) for the 10 mg/kg dose. The trial included 429 patients aged 65 and older (41%), who also showed a 24% survival advantage (HR: 0.76; 95% CI, 0.57–1.02). The 5-year follow-up of the study reported a continued 20% reduction in the risk of death in the over-65 age group [12].

In the OAK trial, atezolizumab was compared to docetaxel [7]. The study included 850 patients, 425 in each arm, with 47% being 65 years or older. The overall survival benefit in the entire study population favored atezolizumab by 27% (HR: 0.73; 95% CI, 0.62–0.87). Interestingly, the survival benefit was slightly better in the 65 and older subgroup, with a 34% reduction in the risk of death (HR: 0.66; 95% CI, 0.52–0.83).

Due to the single-arm nature of our study and the lack of a control group consisting of younger patients, we compared our efficacy data by examining the 65-year-old and younger populations in key studies. In the combined 5-year data from CheckMate 017 and 057, an analysis of 491 patients under 65 years of age showed a median survival of 11.5 months and a 5-year overall survival (OS) rate of 13.4% [11]. Similarly, the 5-year analysis of the Keynote-010 study revealed a median OS of 11.8 months across the entire patient population [12]. This study included 1033 patients with a PD-L1 expression of 1% or greater, of whom 604 were under the age of 65. The hazard ratio (HR) for OS in patients under 65 was 0.62, compared to 0.8 in those aged 65 and older, indicating that while efficacy was observed in the older population, it was somewhat reduced. Lastly, in the OAK study, an analysis of 453 patients under the age of 65 demonstrated a median survival of 13.2 months (10.5 months in the docetaxel arm) [7]. Notably, in contrast to other studies, this study reported a median survival of 14.1 months (9.1 months in the docetaxel arm) for patients over the age of 65, indicating numerically better efficacy compared to the younger population. In our study, we observed a median OS of 23 months and a 5-year OS rate of 18.6%, indicating that the outcomes in our elderly population are also quite robust.

Looking at real-world data and observational studies with similar designs to our study, valuable real-world studies using nivolumab as a single agent in metastatic NSCLC patients who have progressed after at least one line of chemotherapy stand out. In the Italian EAP study by Grossi et al., 371 patients received single-agent nivolumab [13]. Of these patients, 175 (47%) were 65–74, and 70 (19%) were 75 and older. The response rate to nivolumab was 18%, and the disease control rate was 47%, similar to the general population. Although the OS in the 75 and older population was slightly lower at 5.8 months, the 65–74 age group and the general population had similar OS rates of 8 months. In another single-center retrospective study by Galli et al., 290 metastatic NSCLC patients were analyzed [14]. The population aged 70 and older constituted 38% of the patients. The response and OS rates were similar in both the under-70 and over-70 age groups. The results of the French EAP study, which included 902 patients and used nivolumab as a second- or later-line treatment, also showed that age was not a significant factor in determining survival [15]. Jurgens et al. analyzed nivolumab-treated patients in the Canadian population, with 13% of the 472 patients being 70 years or older, and found that OS was 12 months regardless of age group [16]. In another study by Jurgens et al., the Canadian population data from the CM-169 trial were analyzed, focusing on elderly patients. In this study, nivolumab was administered to patients who had previously received chemotherapy, and OS data were examined. Of the 169 patients included, 30% were 70 years or older. OS was 9.1 months in the overall population and 8.0 months in the 70 and older group [17].

Another concern regarding the use of immunotherapy in elderly patients with metastatic lung cancer is the lack of safety data. Immune-related adverse events occur due to activated T cells attacking normal tissues. According to meta-analysis results from previous years, the overall rate of immune-related adverse events with ICI use, regardless of tumor type, ranges from 30 to 65%, with life-threatening grade 3 and above immune-related adverse events occurring in about 5–10% of cases [18,19]. The most commonly affected organs are the endocrine system, skin, colon, liver, and lungs. The most significant concern when using ICIs in elderly patients is that in the event of a grade 3 or 4 adverse event, insufficient baseline organ function may lead to irreversible organ failure and patient loss. In pivotal single-agent immunotherapy trials, no significant differences in toxicity were observed between older and younger populations.

In our study, 45% of our patients experienced toxicity of any grade related to immunotherapy. Consistent with the literature, skin toxicities such as itching and rash were the most commonly observed, followed by thyroid dysfunction and diarrhea. Although 40% of our patients experienced treatment delays due to side effects, no treatment-related mortality was observed.

Similarly, in the CheckMate 171 trial, no differences in the rate of adverse events were observed between younger and older patients, except for mild-grade diarrhea [20]. In the CheckMate 153 trial, a high-grade treatment-related adverse event rate of 6% was observed in both younger and older populations [21]. However, the underrepresentation of elderly patients in clinical trials, the fact that those included tend to be fitter than others in their age group, and the lack of specific studies mean that while the toxicity profile in elderly patients appears similar to that of younger patients, a meta-analysis by Wang et al. highlighted that deaths due to immune-related adverse events were more common in the elderly population [22].

The retrospective nature of our study and the small sample size are among its limitations. Another limitation is including only patients who received nivolumab as second-line or later therapy. Another limitation of our study is that a survival comparison could not be made between patients who received only nivolumab and patients who received nivolumab treatment after chemotherapy. In our country, due to reimbursement policies, we can only use nivolumab as a second-line or later therapy, which we acknowledge as a limitation. We hope this study will provide valuable insights for countries with similar reimbursement constraints. We believe that prospective comparisons between patients receiving ICI after chemotherapy and those receiving ICI treatment directly in future studies will provide more comprehensive clinical data for this patient group. Prospective studies involving more patients are needed to establish the standards for immunotherapy in elderly patients. Additionally, using other immunotherapy agents that we could not use in our study would help increase our knowledge in this area

## 5. Conclusions

Demonstrating the success of oncological treatments in older patients due to accompanying comorbidities poses difficulties compared to younger patient populations because non-malignant mortality also has an impact on survival in this age group. However, our study demonstrated that older patients with advanced NSCLC derived similar benefits from immunotherapy as reported in landmark trials evaluating younger patients. Based on our results, overall survival in this patient population is driven primarily by the number of treatment cycles received and the initial response to therapy rather than chronological age. ICI treatment is generally well tolerated in older patients. Prospective studies are needed to better capture the efficacy and toxicity of immunotherapy among older patients with advanced NSCLC. Such studies will better inform ICI-based treatment decision-making and supportive care interventions for these more vulnerable patients, where the role of a geriatric assessment could be further defined.

## Figures and Tables

**Table 1:** Demographic characteristics of the patients.

Patient Characteristics (*n* = 60)	Category	Statistics
Age (year)	median (IQR, range)	67 (66–71, 65–88)
Sex, *n* (%)	Female	13 (21.7)
	Male	47 (78.3)
Comorbidity, *n* (%)	No	18 (30)
	Yes	42 (70)
Histology, *n* (%)	Adeno ca	27 (45)
	Squamous ca	22 (36.7)
	Mixed	11 (18.3)
Stage at Diagnosis, *n* (%)	Locally	21 (35)
	Metastatic	39 (65)
Number of Metastatic Sites, *n* (%)	Sinle	42 (70)
	Multiorgan	18 (30)
Immunotherapy (IT), *n* (%)	2.line	44 (73.3)
	3–4.line	16 (26.7)
Number of IT Cycles, *n* (%)	<15	40 (66.7)
	≥15	20 (33.3)
Toxicity, *n* (%)	No	33 (55)
	Yes	27 (45)
Treatment Delay, *n* (%)	No	36 (60)
	Yes	24 (40)
Response to Pre-IT CT/RT, *n* (%)	Responder ^a^	22 (36.7)
	Non-responder ^b^	38 (63.3)
IT Response	Responder ^a^	22 (36.7)
	Non-responder ^b^	38 (63.3)
Post-IT progression, *n* (%)	No	26 (43.3)
	Yes	34 (56.7)
Follow-up Duration (months)	median (IQR, range)	23 (19–36, 6–82)
Progression-Free Survival (months)	median (IQR, range)	8 (3–14, 3–35)

IQR, Inter Quantile Range; ^a^, complete or partial response; ^b^, stable disease or progression; IT, immunotherapy; CT, chemotherapy; RT, radiotherapy.

**Table 2:** Variables affecting overall survival.

		Univariable ^a^		Multivariable ^b^	
Patient Characteristics	Category	HR (95% CI)	*p*	HR (95% CI)	*p*
Age (year)	<70	Reference		Reference	
	≥70	1.16 (0.56–2.41)	0.690	1.03(0.37–2.82)	0.958
Sex	Female	Reference		Reference	
	Male	0.99 (0.42–2.29)	0.974	1.34(0.39–4.57)	0.643
Comorbidity	No	Reference		Reference	
	Yes	0.73 (0.33–1.63)	0.447	1.09(0.35–3.41)	0.881
Histology	Adenoca	Reference		Reference	
	Other	1.91 (0.92–3.97)	0.084	5.31(1.74–16.18)	0.003
Stage at Diagnosis	Locally	Reference		Reference	
	Metastatic	1.60 (0.76–3.35)	0.216	13.43(3.69–48.96)	<0.001
Number of Metastatic Sites	Single	Reference		Reference	
	Multiorgan	2.10 (1.03–4.31)	0.042	2.21(0.66–7.38)	0.198
Immunotherapy (IT)	2 line	Reference		Reference	
	3–4 line	1.26 (0.46–3.42)	0.650	1.26(0.46–3.42)	0.870
Number of IT Cycles	<15	Reference		Reference	
	≥15	0.07 (0.02–0.29)	<0.001	0.09(0.01–0.74)	0.024
Toxicity	No	Reference		Reference	
	Yes	1.02 (0.50–2.08)	0.947	1.83(0.61–5.52)	0.285
IT delay	No	Reference		Reference	
	Yes	0.69 (0.33–1.45)	0.326	0.88(0.34–2.24)	0.781
Response to Pre-IT CT/RT	Responder ^c^	Reference		Reference	
	Non-responder ^d^	2.43 (1.05–5.62)	0.039	4.50(1.16–17.55)	0.030
IT response	Responder ^c^	Reference		Reference	
	Non-responder ^d^	3.65 (1.50–8.89)	0.004	6.89(1.30–36.56)	0.023

HR, hazard ratio; ^a^, results were derived from univariable Cox proportional hazards models; ^b^, results were derived from a multivariable Cox proportional hazards model with all the variables listed in the table.; CI, confidence interval; ^c^, complete or partial response; ^d^, stable disease or progression; IT, immunotherapy; CT, chemotherapy; RT, radiotherapy.

**Table 3:** Variables affecting progression-free survival.

		Univariable ^a^		Multivariable ^b^	
Patient Characteristics	Category	HR (95% CI)	*p*	HR (95% CI)	*p*
Age (year)	<70	Reference		Reference	
	≥70	0.69 (0.33–1.44)	0.325	0.77 (0.33–1.79)	0.544
Sex	Female	Reference		Reference	
	Male	1.16 (0.50–2.66)	0.732	0.62 (0.21–1.80)	0.377
Comorbidity	No	Reference		Reference	
	Yes	0.59 (0.29–1.19)	0.141	0.60 (0.27–1.36)	0.219
Histology	Adenoca	Reference		Reference	
	Other	1.33 (0.66–2.65)	0.426	1.76 (0.75–4.14)	0.194
Stage at Diagnosis	Locally	Reference		Reference	
	Metastatic	0.72 (0.37–1.42)	0.340	0.68 (0.26–1.77)	0.427
Number of Metastatic Sites	Single	Reference		Reference	
	Multiorgan	1.47 (0.72–2.97)	0.288	1.85 (0.63–5.43)	0.261
Immunotherapy (IT)	2. line	Reference		Reference	
	3–4. line	1.12 (0.47–2.68)	0.792	1.39 (0.31–6.23)	0.666
Number of IT Cycles	<15	Reference		Reference	
	≥15	0.10 (0.03–0.34)	<0.001	0.13 (0.03–0.54)	0.005
Toxicity	No	Reference		Reference	
	Yes	0.91 (0.46–1.80)	0.785	1.07 (0.45–2.59)	0.874
IT delay	No	Reference		Reference	
	Yes	0.76 (0.37–1.53)	0.439	0.87 (0.40–1.93)	0.739
Response to Pre-IT CT/RT	Responder ^c^	Reference		Reference	
	Non-responder ^d^	1.46 (0.71–3.01)	0.300	0.65 (0.20–2.09)	0.470
IT response	Responder ^c^	Reference		Reference	
	Non-responder ^d^	6.85 (2.59–18.08)	<0.001	7.76 (2.11–28.61)	0.002

HR, hazard ratio; ^a^, results were derived from univariable Cox proportional hazards models; ^b^, results were derived from a multivariable Cox proportional hazards model with all the variables listed in the table; CI, confidence interval; ^c^, complete or partial response; ^d^, stable disease or progression; IT, immunotherapy; CT, chemotherapy; RT, radiotherapy.

**Table 4:** Variables associated with immunotherapy response.

		All	Responder		
Patient Characteristics	Category	*n*	*n* (%)	OR (95% CI)	*p*
Age (year)	<70	39	12 (30.8)	Reference	
	≥70	21	10 (47.6)	2.05 (0.69–6.11)	0.196 ^a^
Sex	Female	13	6 (46.2)	Reference	
	Male	47	16 (34)	0.60 (0.17–2.09)	0.520 ^b^
Comorbidity	No	18	5 (27.8)	Reference	
	Yes	42	17 (40.5)	1.77 (0.53–5.88)	0.350 ^a^
Histology	Adenoca	27	8 (29.6)	Reference	
	Other	33	14 (42.4)	1.75 (0.60–5.14)	0.306 ^a^
Stage at Diagnosis	Locally	21	7 (33.3)	Reference	
	Metastatic	39	15 (38.5)	1.25 (0.41–3.81)	0.694 ^a^
Number of Metastatic Sites	Single	42	14 (33.3)	Reference	
	Multiorgan	18	8 (44.4)	1.60 (0.52–4.95)	0.413 ^a^
Number of IT Cycles	<15	40	6 (15)	Reference	
	≥15	20	16 (80)	22.67 (5.60–91.71)	<0.001 ^a^
Toxicity	No	33	13 (39.4)	Reference	
	Yes	27	9 (33.3)	0.77 (0.27–2.23)	0.628 ^a^
IT delay	No	36	11 (30.6)	Reference	
	Yes	24	11 (45.8)	1.92 (0.66–5.61)	0.229 ^a^
Response to Pre-IT CT/RT	Responder	22	10 (45.5)	Reference	
	Non-responder	38	12 (31.6)	0.55 (0.19–1.64)	0.282 ^a^

OR, odds ratio; CI, confidence interval; ^a^, Pearson chi-square test; ^b^, Fisher’s exact test. IT, immunotherapy; CT, chemotherapy; RT, radiotherapy.

## Data Availability

The raw data supporting the conclusions of this article will be made available by the authors upon request.
